# How do US states define person-centered and family-involved care in assisted living regulations

**DOI:** 10.3389/frdem.2026.1824907

**Published:** 2026-06-10

**Authors:** Paula Carder, Lindsey Smith, Jennifer N. Bunker, Cassandra L. Hua, Erh-Chi Hsu, Isabelle Boun, Olivia M. Ainsworth, Kali S. Thomas, Eric Jutkowitz

**Affiliations:** 1 Institute on Aging, Portland State University, Portland, OR, United States; 2 Oregon Health and Science University Portland State University School of Public Health, Portland, OR, United States; 3 Johns Hopkins University School of Nursing, Baltimore, MD, United States; 4 Zuckerberg College of Health Sciences, University of Massachusetts Lowell, Lowell, MA, United States; 5 School of Public Health, Brown University, Brown University, Providence, RI, United States; 6 University of Minnesota Twin Cities School of Public Health, Minneapolis, MN, United States

**Keywords:** Alzheimer’s disease, assisted living, care partner, long-term care, public policy

## Abstract

**Introduction:**

A large share of assisted living (AL) residents have a diagnosis of dementia or cognitive impairment, and these individuals can benefit from person-centered care and family-involved care. This study examines whether and how states’ AL licenses address these topics.

**Methods:**

This research analyzed a national database of 249 unique licensing requirements that govern 35,602 ALs in all states and associated regulations, including those that govern memory care (MC) services. We reviewed each regulation for the presence of person-centered or family involved care and then used content analysis of license requirements with at least one relevant policy to identify key categories and regulatory specificity.

**Results:**

A larger share of AL with licenses that govern MC services are covered by person-centered care policies (44%) and family-involved policies (62%), compared to AL without MC-specific requirements (20 and 32% respectively). Key categories of person-centered care included staff training, care planning procedures, and social activities; and family-involved care included care planning, direct involvement in providing care, and support for families. Few states’ licenses contained highly specific regulations.

**Discussion:**

This study reveals substantial variation in whether and how states define and regulate person-centered and family-involved care for people living with dementia in AL. MC-specific licenses are roughly twice as likely to require these policies, but fewer than two-thirds of MC-AL communities are covered. State licenses take different approaches to categorizing and specifying these policies. These findings suggest uneven application of core principles of AL associated with quality of life and satisfaction.

## Introduction

People living with dementia face many losses, including their memories, cognitive and physical abilities, and sense of self. Advocates, such as the Pioneer Network,^1^ an early innovator in culture change for people living with dementia, have long promoted person-centered care for those receiving residential long-term services (Fagan, 2003). Relatedly, the importance of family and other care partners to people with dementia is well documented by gerontologists (Gaugler, 2005; Gaugler and Mitchell, 2022), advocates (Fazio et al., 2018a, 2018b) and long-term services professionals (Hayward et al., 2022; Wei et al., 2024). Assisted living (AL), an increasingly important residential setting for older adults living with dementia, was founded on principles like resident choice, dignity, independence, individuality, and privacy (Wilson, 2007, Assisted Living Workgroup, 2003). These concepts aligned with the notion of personhood described in person-centered care approaches (Fagan, 2003; Kitwood, 1997).

Prevalence of dementia is high among AL residents. An estimated 44% of AL residents have dementia^2^ and 70% have some form of cognitive impairment (Zimmerman et al., 2014a, b). Each day, more than 811,000 people reside in one of the 34,000 AL communities in the US (Jutkowitz et al., 2025a,b). Early innovators in this residential long-term care sector promoted consumer-driven and social model principles that underlie person-centeredness (Carder and Hernandez, 2004; Wilson, 2007), but questions have arisen as to whether AL meets this initial promise due to increasing resident acuity, staffing concerns, and inconsistent regulations for residents with dementia (Zimmerman et al., 2024).

Assisted living residences are regulated by states, resulting in considerable differences between and even within states, as most apply more than one license that governs AL (Smith et al., 2021). Examples include variation in state policies that govern MC services (Carder, 2017), regulatory stringency (Temkin-Greener et al., 2021), and regulatory specificity, referring to the amount of detail provided in licensing requirements (Thomas et al., 2021).

### Person-centered care

The heart of person-centeredness includes supporting the individual’s sense of personhood, including the right to self-expression, autonomy, respect and dignity, as well as supportive relationships (Cordero et al., 2026; Kitwood, 1997; Tunalilar and White, 2026). These practices can reduce behavioral symptoms associated with dementia and enhance quality of life for residents of residential long-term care settings (Lee et al., 2022; Davies et al., 2023).

Although organizations and advocates had long promoted person-centered care, in 2014, the Center for Medicare and Medicaid Services^3^ (CMS) required providers that received Medicaid waiver funds for Home and Community-Based Care services to adopt person-centered practices (Zimmerman et al., 2014a,b). A sizeable share of AL communities could be governed by this rule given that 45 states and DC had a mechanism to reimburse AL care with Medicaid funds in 2019 (Gunderson et al., 2025). The spirit of CMS’s definition has been adopted in AL settings, though without state regulations is not enforceable. More recently, CMS developed a comprehensive model of person-centered primary care that supports people living with dementia and their care partners (Jutkowitz et al., 2025a; Jutkowitz et al., 2025b). Together, these federal actions establish expectations across the states. Bridging regulations and advocacy, CMS defines compliance requirements for person-centered care and the Pioneer Network, among other advocates, describe how care should feel to residents, care partners, and staff.

### Family-involved care

Family involvement in the care of older adults has long been the focus of gerontological research, policy and programs. A 2016 National Academies of Science (NAS) report described strategies to identify, assess, and support family caregivers in healthcare service delivery, how to include family caregiver experiences in quality improvement activities and how to train care professionals in person- and family-centered care (National Academies of Sciences, Engineering, and Medicine, 2016). We recognize that the term “family” includes a variety of care partners, such as unpaid kin and non-kin persons, or “chosen” family. We use family here because AL regulations typically use this word to describe individuals involved in residents’ move-in, assessment and service planning processes.

Family involvement in care may be short-term and episodic during acute care events, or as in the case of dementia, ongoing and progressive (Gitlin and Wolff, 2012), leading some caregivers to continue providing support in the community for 8-years after the onset of their relative’s diagnosis (Jutkowitz et al., 2020). Care partners continue to be involved after a relative’s move into nursing homes or another care setting. Families not only contribute to residents’ health through continued emotional support and caregiving responsibilities, but also provide support for AL staff and help guide organizational policies (Gaugler and Mitchell, 2022; Kemp et al., 2013). Effective family participation in caring for their relatives in nursing home settings hinges on consistent communication and trusted partnerships with facility staff, particularly for families of residents with dementia (Gaugler and Mitchell, 2022; Hayward et al., 2022). Family and staff participation in residents’ lives helps maintain and expand residents’ close social relationships both within and outside of the AL context, offering protective social support (Kemp et al., 2018).

This gap between professional ideals, research and AL regulatory requirements motivates the present study. This study examined the role of public policy, through state licensing requirements for MC-AL, in promoting person-centered and family-involved care.

## Methods

We used a mixed-methods approach to study whether and how states specify person-centered and family-involved care of AL licenses that govern MC services. The quantitative component, health services regulatory analysis, answers the question of whether states have the regulations of interest. The results of the preliminary quantitative methods precede the qualitative method, which answers the question of “how” states describe the requirements. We used qualitative, conventional content analysis to identify key categories and regulatory specificity of state licensing requirements (e.g., policies) for person-centered and family-involved care, thus addressing the question of how states describe their policies.

### Health services regulatory analysis

Health services regulatory analysis links license requirements and a national directory of licensed AL to assess regulatory coverage at the state, community-, and bed- level (Smith et al., 2021). This quantitative approach counts the number of AL residences and beds governed by selected policies. The dataset created by our team (Thomas et al., 2018) includes all regulations for all 249 license types applied to AL communities in all states, operating from 2018 to 2023. Regulations were sourced using Westlaw and NexisUni.

Most states have more than one license applicable to AL. Regulations might include MC-specific requirements for a variety of provisions such as training staff in the care of residents with dementia. Some licenses do not specify care requirements, and instead require AL that provide MC services to “disclose” various types of information to licensing agencies. A state could require AL to disclose the type of training staff will complete; some states use both requirements and disclosure, and a few use disclosure only. For this analysis, we compared states with MC-specific AL regulations and/or disclosure, henceforward referred to as MC-AL, to states that only required disclosure of MC services (e.g., no MC-specific requirements), which we refer to as general-AL. We answered two policy questions: Does the license require person-centered care? Does the license specify family involvement in the care of residents with dementia?

Our interdisciplinary team included three faculty members (two with doctoral training in policy and one in gerontology), one doctoral student with master’s-level clinical training in nursing, one master’s-level student in public policy, two master’s-level students in public health, and three undergraduate research assistants. For each question, two members of our team of 10 coders independently read the text for each license and recorded whether the answer to the question was “yes,” “no,” or the topic was not addressed; authors 1, 2 and 4 reviewed and resolved differences. To increase coding reliability, our team met weekly to discuss interpretations of both the questions and policy texts. See Supplementary Table 1 for coding instructions.

All coding was combined using R statistical environment to create a longitudinal dataset of 576,880 records, each indicating the presence or absence of a specified regulatory requirement for an AL license type, per year. For this analysis, we include regulations active as of 2023.

### Conventional content analysis of AL license requirements

The primary goal of our qualitative, conventional content analysis (Hsieh and Shannon, 2005; Ortiz et al., 2021) was to answer the question of how states’ MC-AL licenses (e.g., excluding general-AL) describe person-centered and family-involved care policies, focusing on the licenses identified during the quantitative approach described above. This approach focuses on the context of regulatory requirements (Ortiz et al., 2021), in addition to the presence of requirements. Our rationale for focusing on MC-AL is that, based on our prior research, this license type would be most likely to include specific provisions related to the care of residents with dementia.

Conventional content analysis includes developing codes from the data (regulatory documents), defining code meanings (see Supplementary Table 2), attaching codes to text, developing key categories and comparing findings to existing literature. We used Atlas.ti software to facilitate this process. To identify sections of text regarding person-centeredness, we searched for -centered, −directed, person-, individualized, and consumer- in addition to reviewing all sections about assessment and care planning. For family-involved care, we read the text of each license and used word search terms such as family, relative, kin and representative to identify relevant text. For memory care-specific policies, we searched for the terms dementia, cognitive, Alzheimer, and memory. We verified the latter using our dataset of AL licenses (described above), including the MC licensing term or policy reference used by each state.

Our qualitative team read license texts and applied codes to relevant sections based on our search terms. This team included two faculty members with graduate training in gerontology and qualitative methods (including Author 1), two doctoral students (sociology and community health) with qualitative methods training and two undergraduate students (public health, aging services). Data quality efforts included initial and ongoing training by the first author and regularly scheduled team meetings. As with the quantitative approach above, each license was reviewed by two coders and discrepancies were compared and resolved in team meetings and by the first author.

After completing this phase of coding, we reviewed output from Atlas.ti to identify key categories and regulatory specificity of person-centered and family-involved care of residents with dementia. We limited the search to sections of text identified as MC-specific, as described above. For the purpose of this analysis, we define a “key category” as one that provides sufficient regulatory specificity to understand the state’s intent. Examples of highly specific regulations, defined as licenses with comprehensive, detailed provisions that can inform future state policies, are included in the Supplementary materials.

## Results

### Quantitative results

**Figure 1:**
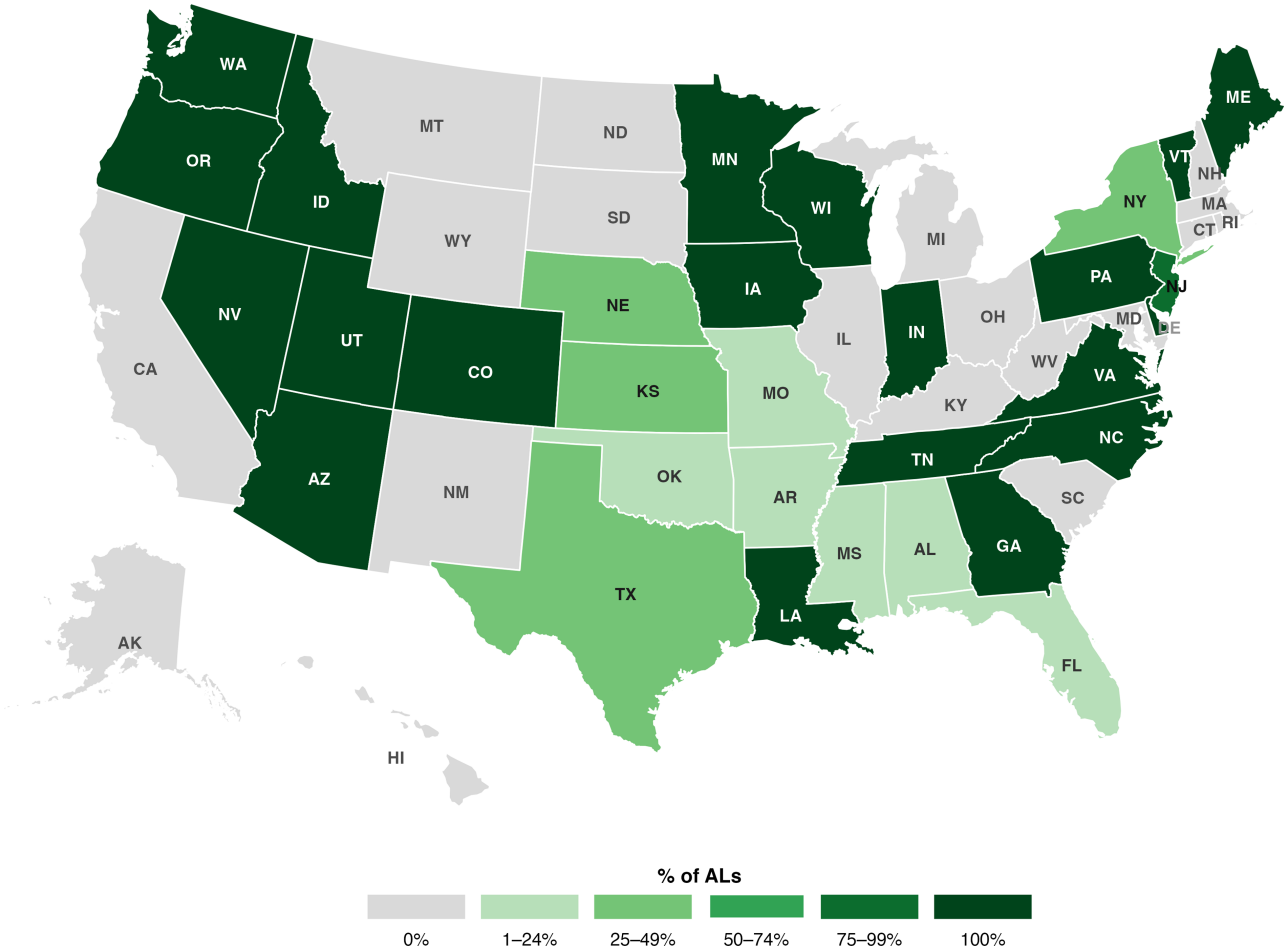
Percentage of ALs with a family involvement policy for residents with dementia by state, 2023. % = ALs with policy/total ALs in state. States in grey = 0% coverage.

**Figure 2:**
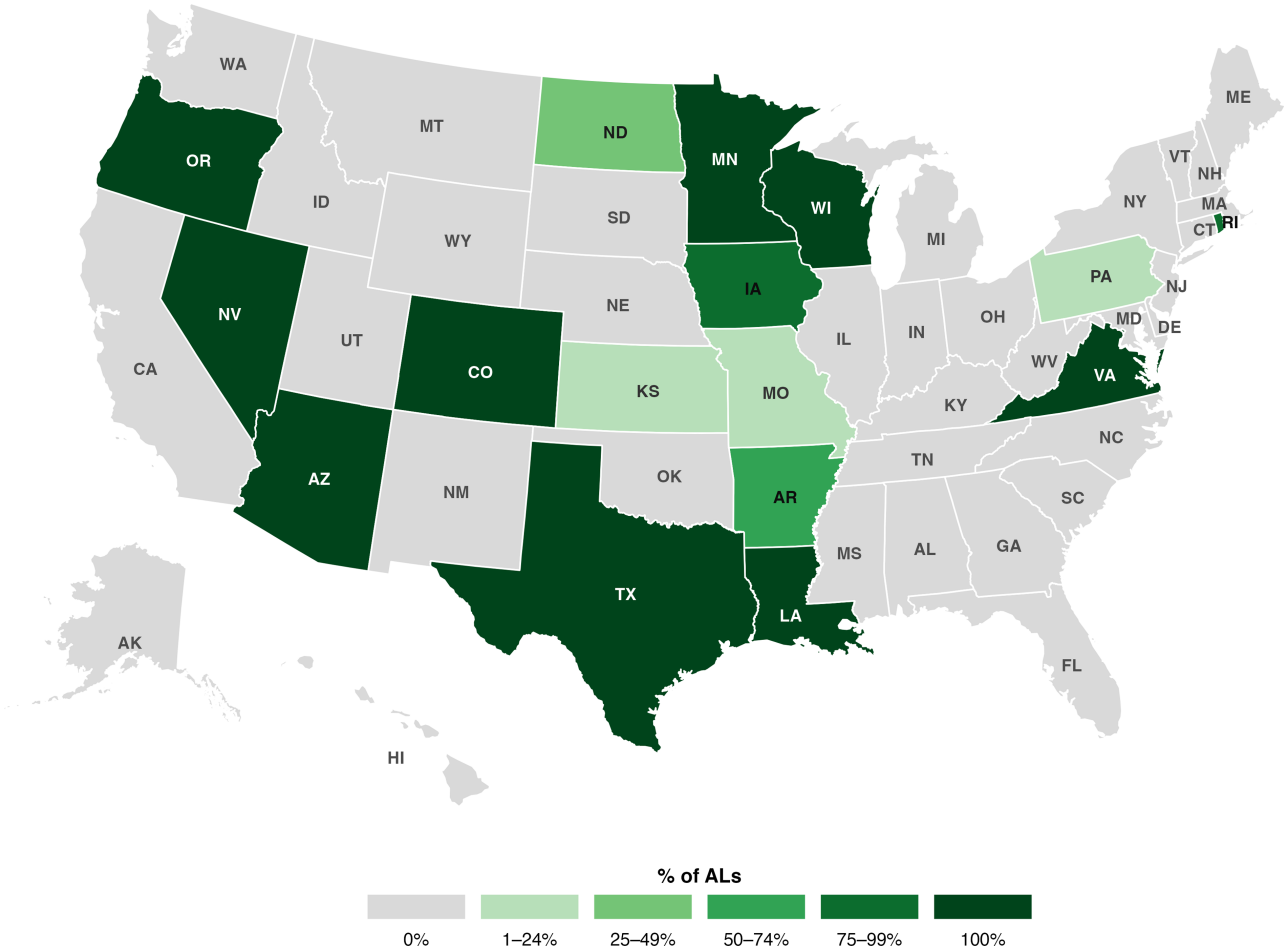
Percentage of ALs required to have a person-centered or person-directed care policy by state, 2023. % = ALs with policy/total ALs in state. States in grey = 0% coverage.

The sample included 249 AL licenses in all states, governing 35,602 ALs in 2023. General-AL communities accounted for twice as many MC-AL beds. A larger share of MC-AL residences are governed by person-centered care policies, compared to general-AL (44%, *n* = 3,348 MC-ALs versus 20%, *n* = 5,698 ALs), with a similar trend for MC-AL governed by family involvement policies (62% *n* = 4,722 MC-ALs versus 34% *n* = 9,506 ALs) (Table 1). The percent of AL communities with a family-involvement policy for residents with dementia or for AL communities required to have a person-centered care policy are presented in Figures 1, 2, by state. The maps indicate, with darker shading, the share of AL communities covered by the policy, while the lightest color (gray) depicts states for which the policy was not present in licensing requirements.

**Table 1:** Policy coverage at assisted living and assisted living bed-level, stratified by type, 2023.

Assisted living type	General AL (*n* = 28,312 ALs; *n* = 954,841 beds)	Memory care AL (*n* = 7,659 ALs; *n* = 460,044 beds)
Require person-centered care policy		
# of ALs Covered (%)	5,698 (20%)	3,348 (44%)
# of AL Beds Covered (%)	191,257 (20%)	190,778 (41%)
Have family involvement policy regarding residents with dementia		
# of ALs Covered (%)	9,506 (34%)	4,722 (62%)
# of AL Beds Covered (%)	400,623 (42%)	291,574 (63%)

*MC is defined as (1) States with MC regulations and disclosure OR (2) States without regulations but required disclosure if they claim to provide MC services.

### Qualitative results

We analyzed the requirements for state licenses described above, and identified key categories and examples of specific MC-AL license requirements. Key categories that describe person-centered care of residents with dementia included staff training, care/service planning procedures, and social activities (Supplementary Table 3). Other topics were addressed but lacked specificity, including built environment, food and culturally-relevant care, and were not identified as key categories.

Key categories associated with family-involved care of AL residents with dementia included care/service planning, family provision of care (e.g., medication administration, end of life) and family supports (e.g., councils) (Supplementary Table 3). Some MC-AL license requirements addressed family involvement in the context of resident rights (e.g., to privacy and visiting), legal aspects (e.g., lease, payments), communication from AL to family, and staff training; however, these had low specificity and were not included in key categories.

We observed varying degrees of specificity in the regulations (Table 2). New York’s requirements for “Special Needs Assisted Living Residences,” an example from the care/service planning category, provides a very specific example of involving family in care/service planning for residents with “special needs,” including those with dementia:

**Table 2:** Selected examples of person-centered and family-involved license requirements for MC-AL residences.

Example of specific MC-AL staff training in person-centered care
Colorado assisted living residence 1,011–1:7–25. Secure environment 25.15 Within sixty (60) days, the assisted living residence shall provide each staff member a minimum of six (6) hours of general training and education on providing care and services for residents with dementia/cognitive impairment. (A) The training may be provided over several sessions. (B) The training shall be provided through structured, formalized classes, correspondence courses, competency-based computer courses, training videos, or distance learning programs. (C) The training content shall be provided or recognized by an academic institution, a recognized state or national organization or association, or an independent contractor or group that emphasizes dementia/cognitive impairment care. (D) The training shall cover, at a minimum, the following topics: (1) Information on disease processes associated with dementia and cognitive impairment, including progression of the diseases, types and stages of memory loss, family dynamics, behavioral symptoms and limitations to normal activities of daily living; (2) Information on non-pharmacological techniques and approaches used to guide and support residents with dementia/ cognitive impairment, wandering, and socially challenging behavioral expressions of need or distress; (3) Information on communication techniques that facilitate supportive and interactive staff-resident relations; (4) Positive therapeutic approaches and activities such as exercise, sensory stimulation, activities of daily living and social, recreation, and rehabilitative activities; (5) Information on recognizing physical symptoms that may cause a change in dementia/cognitive impairment such as dehydration, infection, and swallowing difficulty; along with individualized approaches to assist or address associated symptoms such as pain, decreased appetite and fluid intake, and/or isolation; and (6) Benefits and importance of person-centered care planning and collaborative approaches to delivery of care.

Examples of specific MC-AL license requirement for family-involvement
Iowa 481–57.6 (135C). 57.6 (1) Memory care. Training shall include the following topics: (II, III) An explanation of Alzheimer’s disease and related disorders, including symptoms, behavior and disease progression; Skills for communicating with persons with dementia; Skills for communicating with family and friends of persons with dementia; An explanation of family issues such as role reversal, grief and loss, guilt, relinquishing the caregiving role, and family dynamics
Florida 59A-36.021. Extended congregate care services. Family or friends must be encouraged to provide supportive services for residents. The facility must provide training for family or friends to enable them to provide supportive services in accordance with the resident’s service plan.

### Case management in special needs assisted living

(i) In addition to the case management services required by subdivision (i) of this section, the operator shall assist the special needs assisted living resident to maintain family ties by assisting residents’ family members and representatives to: (a) adjust to and remain involved with the resident’s initial placement and continued residence in the special needs assisted living residence; (b) establish, operate, and maintain individual and collective methods or recommendations for change or improvement in residence operations and programs, regarding both individual and congregate resident-related issues; (c) remain active in the care planning process for the resident; and remain informed in a timely manner about significant issues regarding the resident’s care and supervision needs and changes made to the care plan. [New York. Resident services, 10 NY ADC 1001.10].

Oregon’s memory care license, included in Supplementary Table 3, describes staff training in person-centered care in this way:

*Person centered care promotes a positive relationship between the resident and staff which is accomplished by staff being knowledgeable about the resident’s life story, routines, and habits, and incorporating that information into the individual’s daily care and activities. (1) The licensee is responsible for the operation of the memory care community and the provision of person centered care that promotes each resident’s dignity, independence, and comfort. This includes the supervision, training, and overall conduct of the staff. [OAR 411-057-0140.]*

Examples of license requirements that lacked specificity, include search terms that were mentioned in bullet lists or brief statements: Maine’s requirements for pre-service training for specialty care units, describes only, “Family Issues.” [Maine 10-144 CMR Ch. 113, LIIIR § 6.8.6].

## Discussion

This study describes regulatory coverage (e.g., scope) and key categories of person-centered and family-involved care requirements in state-level licenses that govern general and MC-AL services. States take different regulatory approaches to defining and overseeing memory care (Carder, 2017), though many license or certify memory care in AL and nearly every state has at least one MC-specific policy (Smith et al., 2021). Our novel health services regulatory approach of accounting for within-state variation in license requirements at facility- and bed-levels illustrates the prevalence of residents who live in an AL with one of these policies. We inform policymaking by describing key categories associated with MC-AL requirements for person-centered and family-involved care, and provide examples of specific policies that govern these licenses in some states.

Despite widespread endorsement of person-centered and family-involved are for people living with dementia, little has been written about how state AL regulations address these topics for MC-AL settings. A review of AL staff training requirements found that over half of states prioritize safety-related training—such as CPR certification, infection control, and fire emergency protocols—while fewer address person-centered topics: only 22 states required training in consumer rights and only 10 required culturally relevant training (Kelly et al., 2020).

### Person-Centered care in AL

Based on quantitative findings, this analysis indicates that person-centered care policies were more often present in MC-AL compared to general AL. The key categories within the MC-AL licenses described staff training and care planning requirements. For example, Colorado’s “secure environment” rules indicate that staff should use “collaborative approaches to delivery of care” (see Table 1). The importance of staff in the well-being and satisfaction of AL residents is well documented, and staff training on person-centered care was the item most recommended by a consensus paper on AL medical and mental health care (Zimmerman et al., 2022b). While most states described training for direct care staff, a few, including Oregon, require all “memory care community” staff to be trained in person-centered care (see Supplementary Table 3).

Person-centered care is also reflected in AL professional communications, including the National Center for Assisted Living^4^ and Argentum^5^. The Assisted Living Workgroup (ALW) identified “resident-centered services” as a core principle for states’ AL regulations and other policies (Assisted Living Workgroup, 2003) and the Alzheimer’s Association defines practice recommendations for person-centered care (Fazio et al., 2018b).

Measuring whether person-centered processes improve quality of life is important, and several dimensions of person-centeredness and strategies for assessing outcomes have been identified (Roberts and Abery, 2023; Tunalilar and White, 2026; Zimmerman et al., 2015). Recent work focuses on person-centered care measures linked to meaningful outcomes, including dimensions of well-being for people living with dementia (Zimmerman and Fazio, 2022).

### Family-involved care in AL

Categories of family involvement identified in this study align with other research. For example, residents living with dementia and other forms of cognitive impairment, especially those who cannot convey their intent, benefit from family and other care partner input into decisions regarding care planning, end of life care, legal decisions and meaningful activities (Fazio et al., 2018a,b). The Covid pandemic prompted innovative strategies for engaging families of residents in residential long-term care settings including remote family involvement in care planning, technology-supported social visits, virtual health promotion for residents’ families, and structured communication between staff and families (Bowers et al., 2021; Dys et al., 2021). States and AL providers may build on these innovations by adopting policies that support remote care planning, visits and communication.

Assisted living and other residential long-term care settings can and do support residents’ care partners by including them as partners and training staff in family communication methods. Recent research described families and other care partners as “#morethanavisitor” who consider themselves as team members, volunteers, and advocates for change (Kemp, 2021; Klostermann and Funk, 2024). Florida’s extended congregate care rules are unique for requiring family or friends to “be encouraged to provide supportive services for residents” (see Table 2).

Assisted living residents’ care partners benefit from training in end of life care and emotional supports when their relative receives hospice services (Guo et al., 2025; Guo et al., 2023). We observed that a small number of MC-AL requirements for staff training include communication between staff and families, and understanding the needs of families whose relatives have dementia. For example, Iowa’s memory care rules require staff to receive training in “skills for communicating with family and friends and an explanation of family issues such as role reversal, grief and loss, guilt, relinquishing the caregiving role, and family dynamics” (see Table 2).

Adult foster home operators in Oregon, a setting licensed as AL in some states, reported that residents’ families participated by providing care, including administering medications (Tunalilar, 2024). Similarly, we observed that some AL licenses, including those in Florida and Washington, describe family involvement in care, such as medication administration, in additional to participating in social activities.

Poor communication between staff and resident’s care partners can result in conflict and low satisfaction (Bauer and Nay, 2011; Kemp et al., 2009; Kemp et al., 2013). Existing research suggests opportunities for AL policymakers and providers to consider care partners as more than “unpaid” or “informal” caregivers and whether current training requirements prepare staff to understand the changing needs of residents living with dementia, and their families.

### Regulating assisted living

How best to regulate AL has been a topic of concern to policymakers, providers, advocates and consumers since its founding, with occasional calls from the US Congress and journalists for federal oversight rather than the existing “patchwork” of state regulations (Smith and Xu, 2021; Rau, 2018; Weil and Rich, 2023). Regulatory patchwork can result in uncertainty, inconsistency and safety gaps, but can also reflect legitimate expressions of state rights and local context (Price, 2017).

The regulatory variation described in this study is not new. Over 20 years ago, the Assisted Living Workgroup (Assisted Living Workgroup, 2003) recommended guidelines for AL regulations to the US Senate Special Committee on Aging. The guidelines, while describing person-centered and family-involved care, also reflected disagreement among the 48 member organizations. These differences of opinion as to whether the recommendations should be “prescribing, in detail, the processes that a state should require of AL residences” and that “state governments should be granted regulatory flexibility” (Assisted Living Workgroup, 2003, page 148) illustrate long-standing tensions. The regulatory variation described in this and other studies (Smith et al., 2021; Temkin-Greener et al., 2021; Thomas et al., 2021) can be understood in the context of regulatory silence and regulatory specificity. Silence, reflecting the absence of a policy, might be due to a lack of evidence about what works or to concerns about economic costs of implementing or overseeing a policy (Bradford, 2024).

While potentially promoting flexibility, low regulatory specificity, including vague policies or silence, can leave entities uncertain about regulators’ expectations (Adams, 2023; Dys et al., 2021). Regulatory specificity describes policies that are highly detailed and specific about licensors’ expectations. But achieving regulatory specificity is difficult when parties lack agreement (Spiller, 1996), as in the above example from the ALW report (2003). States like Oregon and Georgia (see Supplementary materials) demonstrate that highly detailed, actionable rules are achievable, possibly serving as models for other states.

### Do AL policies lag practice?

This study suggests that states’ AL regulations for person-centered and family- involved care in AL lag behind practice ideals widely endorsed by advocates and researchers (Fazio et al., 2018a; Fazio et al., 2018b; Kitwood, 1997; Gaugler and Mitchell, 2022; Zimmerman et al., 2022a; Zimmerman et al., 2024). One solution could be found in states that adopt a public health approach to dementia that could benefit AL professionals and policymakers, as well as residents and their care partners (Kaskie et al., 2025). Examples include collaborative actions among advocacy organizations, legislative and executive staff, elected officials, and senior housing professionals who collectively inform policy agendas concerning the needs of residents with dementia and their care partners. Based on this and related studies, such efforts can advance policy by addressing the variable coverage in AL licensing requirements that specifically describe person-centered and family-involved care for all residents, including those living with dementia.

## Limitations

States might have modified license requirements since 2023 and states might include person-centered or family-involved concepts without using any of the key terms used by our team to search over 10,000 pages of text. This study cannot account for innovations in how AL communities elect to implement policies which may exceed requirements in practice. Senior housing professional literature offers examples of providers that do so. Future research could build on these findings by identifying whether states with the identified policies enforce them through deficiencies, and by assessing resident, family and staff satisfaction with person-centered and family-involved care.

Future research could develop a specificity score and examine resident outcomes, as our team’s prior study did to assess specificity of AL staffing requirements (Thomas et al., 2021). Other potential directions include assessing whether regulatory enforcement actions address failure to implement person-centered and family involved care policies, and examining how COVID-era innovations (technology, family care partnerships) can be embedded in future policy.

## Conclusion

This study reveals substantial variation in whether and how states define and regulate person-centered and family-involved care in licenses that govern MC-AL. While MC-specific licenses are roughly twice as likely to require these policies, fewer than two-thirds of MC-AL communities are covered. Given that an estimated 70% of AL residents have some form of cognitive impairment (Zimmerman et al., 2014a, 2014b), expanding specific person-centered and family-involvement requirements to general AL licenses represents the clearest policy opportunity to improve quality of life across the sector. It is a widely held belief that person-centered and family-involved care are not just amenities, but rather essential to maintaining the dignity, personhood, and quality of life that advocates, researchers, and professionals have long argued every person living with dementia deserves (Kitwood, 1997; Fazio et al., 2018a; Fazio et al., 2018b). Given the lack of literature on the presence of person-centered and family involved care policies in AL on residents’ quality of life, this research can inform future work that assesses the outcomes of these policies in the identified states.

## Data Availability

The data analyzed in this study is subject to the following licenses/restrictions: these datasets, or components of them, may be available from the study PIs upon request. Requests to access these datasets should be directed to kali.thomas@jhu.edu or jutko001@umn.edu.

## Footnotes

1
https://www.pioneernetwork.org/

2 National Post-Acute and Long-Term Care Study (2022). Table 4: Post-acute and Long-term Care User Demographic and Health-related Characteristics: United States. https://www.cdc.gov/nchs/npals/webtables/overview.htm Accessed 2/28/26.

3
https://www.medicaid.gov/medicaid/home-community-based-services/home-community-based-services-guidance-additional-resources/home-community-based-services-final-regulation

4
https://www.ahcancal.org/Assisted-Living/About-NCAL/Documents/GPQualityinAssistedLiving.pdf

5
https://www.argentum.org/person-centered-dementia-care-in-senior-living/
