# Supplementary material for: How do US states define person-centered and family-involved care in assisted living regulations
Source: Front Dement. 2026 Jun 10;5:1824907. doi: 10.3389/frdem.2026.1824907 (PMC13291062; doi:10.3389/frdem.2026.1824907)
Supplement: Supplementary file 1 [file Data_Sheet_1.PDF]

Supplementary Material

Supplementary Table 1. Codebook for health services regulatory coding

**Policy question**	**Coding instructions**
Does the license require the facility to have a policy or procedures specific to person-centered or person-directed care?	Look for "person-centered" and "person-directed". Note that the hyphen might not be used. It might be in the definitions or resident services/care planning section. Code YES only if one of these phrases is used.
Does the license have a policy regarding family involvement in care for residents with dementia?	States might state that facilities should/must have a policy describing how and if families should be involved in care planning, responding to behaviors, and other aspects of resident care besides payment. The rules might or might not give specific examples of how to involve families. Treat "family" broadly, including friends, or other terms states use to imply family. Code YES if the rules state that facilities should involve families in care or service planning and related activities, but not simply as an emergency contact. We are looking for involvement.

Supplementary Table 2. Codebook for conventional content analysis

Code Name	Definition
Family_general	Any mention of residents' family members. Some states will be inclusive of non-kin family, including "care partner" or similar. Search for family, relative, kin, significant other, children, spouse, resident's representative, responsible party/person.
Family involved in care planning	Specific description of family role in care or service planning. Could be as brief as a policy or detailed description of how to involve family, including end of life. Does not include leases, contracts or payment of fees.
Staff training-family specific	Does staff training refer to families, as in, communicating with, understanding their feelings, etc.
Staff training-dementia specific	Use for any text that describes staff training in care for people with dementia. This could be in general AL as well as in "specialty" care regulations. Double code with other staff training codes as relevant.
Family_dementia specific	This will be double coded with the other family codes, to capture any dementia specific care planning or general mentions of family (e.g., family council).
Staff training-topics	Many states list the specific topics (e.g., understanding aging, assisting with personal care) required to be addressed in training staff.
Person-centered care	States might describe resident- or person-driven, resident- or person-directed, or similar language. The text should describe how to find out what makes each individual resident unique, their preferences, routines, and activities that allow them to feel like themselves.
Memory care definition	If the rules define memory care in the definitions or purpose section, please code. Might be referred to as special/specialty care, dementia care or similar.
Disclosure of MC services	Some states require AL to "disclose" whether they offer memory/dementia or special services for people with ADRD. The text will use: disclose, disclosure, market/ing, or advertise/advertising.

	Code the entire section, if located.

Supplementary Table 3. Selected examples of licensing requirements with highly specific license requirements that govern memory care-AL

**Person-centered care for residents with dementia**	
**Key category**	**Regulatory text**
Care/service planning	Oregon administrative rules that govern person-centered care in Assisted Living and Residential Care facilities (OAR 411-054), including Endorsed Memory Care Communities (OAR 411-057)<br>**OAR 411-054-0005. Definitions**<br>(62) “**Person-Centered Service Plan**” means the details of the supports, desired outcomes, activities, and resources required for an individual to achieve and maintain personal goals, health, and safety, as described in OAR 411-004-0030.<br>(a) FOR INDIVIDUALS RECEIVING MEDICAID. The person-centered service plan coordinator completes the person-centered service plan.<br>(b) FOR NON-MEDICAID INDIVIDUALS. The person-centered service plan may be completed by the resident, and as applicable, the representative of the individual, and others as chosen by the individual. The licensee may assist non-Medicaid individuals in developing person-centered service plans when no alternative resources are available. The elements of the individual's person-centered service plan may be incorporated into the resident's care plan.<br>(63) “**Person-Centered Service Plan Coordinator**” means a:<br>(a) Resident's AAA or APD case manager assigned to provide case management services or person-centered service planning for and with individuals; or<br>(b) Person of the individual's choice for individuals who pay privately.<br>411-054-0027. Resident Rights and Protections<br>HCBS Rights<br>(B) The freedom and support to access food at any time.<br>(C) To have visitors of the resident's choosing at any time.<br>(D) Choose a roommate when sharing a bedroom.<br>(E) Furnish and decorate the resident's bedroom according to the Residency Agreement.<br>(F) The freedom and support to control the resident's schedule and activities.<br>(b) The rights described in (B) through (F) of this section must meet the requirements set forth in OAR 411-054-0038 and shall not be limited without the informed, written consent of the resident or the resident's representative, and approved by the person-centered service plan coordinator.<br>**411-054-0036. Service Plan -- General**

	(1) If the resident has **a Person-Centered Service Plan** pursuant to 411-004-0030, the facility must incorporate all elements identified in the person-centered service plan into the resident's service plan.<br>(2) SERVICE PLAN. The service plan must reflect the resident's needs as identified in the evaluation and include resident preferences that support the principles of dignity, privacy, choice, individuality, and independence.<br>(a) The service plan must be completed:<br>(A) Before resident move-in, with updates and changes as appropriate within the first 30-days; and<br>(B) Following quarterly evaluations.<br>(b) The service plan must be readily available to staff and provide clear direction regarding the delivery of services.<br>(c) The service plan must include a written description of who shall provide the services and what, when, how, and how often the services shall be provided.<br>(d) Changes and entries made to the service plan must be dated and initialed.<br>(e) When the resident experiences a significant change of condition the service plan must be reviewed and updated as needed.<br>(f) A copy of the service plan, including each update, must be offered to the resident or to the resident's legal representative.<br>(g) The facility administrator is responsible for ensuring the implementation of services.<br>(3) SERVICE PLAN REQUIREMENTS BEFORE MOVE-IN.<br>(a) Based on the resident evaluation performed before move-in, an initial service plan must be developed before move-in that reflects the identified needs and preferences of the resident.<br>(b) The initial service plan must be reviewed within 30-days of move-in to ensure that any changes made to the plan during the initial 30-days, accurately reflect the resident's needs and preferences.<br>(c) Staff must document and date adjustments or changes as applicable.<br>(4) QUARTERLY SERVICE PLAN REQUIREMENTS.<br>(a) Service plans must be completed quarterly after the resident moves into the facility.<br>(b) The quarterly evaluation is the basis of the resident's quarterly service plan.<br>(c) If the resident's service plan is revised and updated at the quarterly review, changes must be dated and initialed, and prior historical information must be maintained.<br>(5) SERVICE PLANNING TEAM. The service plan must be developed by a Service Planning Team that consists of the resident, the resident's legal representative, if applicable, any person of the resident's choice, the facility administrator or designee and at least

	one other staff person who is familiar with, or who is going to provide services to the resident. Involved family members and case managers must be notified in advance of the service-planning meeting.<br>(a) As applicable, the Service Planning Team must also include:<br>(A) Local APD or AAA case managers and family invited by the resident, as available.<br>(B) A licensed nurse if the resident shall need, or is receiving nursing services or experiences a significant change of condition as required in 411-054-0045(1)(f)(D) (Resident Health Services).<br>(C) The resident's physician or other health practitioner.<br>(b) Each resident must actively participate in the development of the service plan to the extent of the resident's ability and willingness to do so. If resident participation is not possible, documentation must reflect the facility's attempts to determine the resident's preferences.<br>(6) RISK AGREEMENT. When a resident's actions or choices pose a potential risk to that resident's health or well-being, the facility may utilize a risk agreement to explore alternatives and potential consequences with the resident.<br>(a) The facility must identify the need for and develop a written risk agreement following the facility's established guidelines and procedures. A risk agreement must include:<br>(A) An explanation of the cause of concern;<br>(B) The possible negative consequences to the resident or others;<br>(C) A description of the resident's preference;<br>(D) Possible alternatives or interventions to minimize the potential risks associated with the resident's current preferences and actions;<br>(E) A description of the services the facility shall provide to accommodate the residents' choice or minimize the potential risk; and<br>(F) The final agreement, if any, reached by all involved parties, must be included in the service plan.<br>(b) The licensing policy analyst must be consulted and alternatives reviewed before the resident signs the agreement.<br>(c) The facility must involve the resident, the resident's designated representative, and others as indicated, to develop, implement, and review the risk agreement. The resident's preferences shall take precedence over those of a family member.<br>(d) A risk agreement shall not be entered into or continued with, or on behalf of, a resident who is unable to recognize the consequences of their behavior or choices.<br>(e) The risk agreement must be reviewed at least quarterly.<br>**411-054-0038. Individually-Based Limitations**<br>(3) An individually-based limitation must be supported by a specific assessed need and documented in the person-centered service plan by completing and signing a program approved form

	documenting the consent to the appropriate individually-based limitation. The form identifies and documents, at a minimum, all of the following requirements:<br>(a) The specific and individualized assessed need justifying the individually-based limitation.<br>(b) The positive interventions and supports used prior to any individually-based limitation.<br>(c) Less intrusive methods that have been tried but did not work.<br>(d) A clear description of the limitation that is directly proportionate to the specific assessed need.<br>(e) Regular collection and review of data to measure the ongoing effectiveness of the individually-based limitation.<br>(f) Established time limits for periodic reviews of the individually-based limitation to determine if the limitation should be terminated or remains necessary. The individually-based limitation must be reviewed at least annually.<br>(g) The informed consent of the individual or, as applicable, the legal representative of the individual, including any discrepancy between the wishes of the individual and the consent of the legal representative.<br>(h) An assurance that the interventions and support do not cause harm to the individual.<br>(i) If using a restraint, a facility must meet the requirements of OAR 411-054-0060.
Staff training	**Oregon Assisted living**<br>**411-054-0070. Staffing Requirements and Training**<br>(D) Information concerning specific aspects of dementia care and ensuring the safety of residents with dementia, including, but not limited to, how to:<br>(i) Identify and address pain.<br>(ii) Provide food and fluids.<br>(iii) Prevent wandering and elopement.<br>(iv) Use a person-centered approach.<br>**Endorsed Memory Care Communities**<br>411-057-0110. Definitions<br>Unless the context indicates otherwise, the following definitions and the definitions in OAR 411-054-0005 relating to Residential Care and Assisted Living Facilities and OAR 411-085-0005 relating to Nursing Facilities apply to these rules.<br>(16) “Endorsement” means the community has met the requirements to provide specialized services in a memory care community and the requirements for the community's underlying license. An endorsement does not constitute a recommendation of any memory care community by the Division.

	(23) “Person Centered Care” is an approach that supports a resident to direct their own care by focusing on what is important to them while taking into account all the factors that impact their life. Person centered care promotes a positive relationship between the resident and staff which is accomplished by staff being knowledgeable about the resident's life story, routines, and habits, and incorporating that information into the individual's daily care and activities.<br>411-057-0140. Responsibilities of Administration<br>(1) The licensee is responsible for the operation of the memory care community and the provision of person centered care that promotes each resident's dignity, independence, and comfort. This includes the supervision, training, and overall conduct of the staff.<br><br>(5) In addition to the policies and procedures required in the licensing rules for the facility, the memory care community licensee must develop and implement policies and procedures that address:<br>(a) Philosophy of how services are provided based upon the memory care community's values, mission, and the promotion of person centered care and how it shall be implemented;<br>(b) Evaluation of behavioral symptoms and design of person centered supports for intervention plans;<br>(c) Wandering and egress prevention that provides detailed instructions to staff in the event a resident elopes;<br>(d) Assessment of residents for the use and effects of medications including psychotropic medications;<br>**411-057-0150. Staffing Requirements**<br>STAFFING REQUIREMENTS. The facility must provide residents with dementia trained staff who have been instructed in the person centered care approach. Prior to providing care and services to residents all staff must receive training as required in OAR 411-057-0155.
Staff training	Colorado Assisted Living Residence<br>1011-1:7-25. Secure Environment<br>25.12 The assisted living residence shall have a policy and procedure regarding the training of staff who provide services in a secure environment. The policy shall include, at a minimum, information on the appropriate staff response when there is a missing resident or resident incident/altercation, along with distribution of staff when responding to such an event to ensure that there is sufficient staff presence for the continued supervision of other residents.<br>25.13 In addition to the training requirements in Part 7.9, staff assigned to a secure environment shall receive training and

	education on assisted living residence policies and procedures specific to the secure environment resident care, services, and protections. Such training shall include, at a minimum, the following:<br>(A) Information on the secure environment that identifies and describes the areas where residents have free passage, where passage may be restricted, and where passage is prohibited;<br>(B) Information regarding the current mobility status of all residents so that staff are prepared to successfully evacuate all residents in the event of an emergency;<br>(C) Information on the location of the storage area which is not accessible to residents including a description of what items or contents are required to be kept in the storage area; and<br>(D) Information on the equipment and devices used to secure the environment, including how to override or disarm such devices, along with expectations for response if staff are alerted to an alarm.<br>25.14 Before a staff member is allowed to work independently in the secure environment, the assisted living residence shall provide each staff member with training and education on the provision of care and services for the specific population in the assisted living residence.<br>(A) At a minimum, the individual shall be trained on the care plan for each resident to which the individual could provide care given the staff member's assigned duties and responsibilities. Such training shall be documented.<br>25.15 Within sixty (60) days, the assisted living residence shall provide each staff member a minimum of six (6) hours of general training and education on providing care and services for residents with dementia/cognitive impairment.<br>(A) The training may be provided over several sessions.<br>(B) The training shall be provided through structured, formalized classes, correspondence courses, competency-based computer courses, training videos, or distance learning programs.<br>(C) The training content shall be provided or recognized by an academic institution, a recognized state or national organization or association, or an independent contractor or group that emphasizes dementia/cognitive impairment care.<br>(D) The training shall cover, at a minimum, the following topics:<br>(1) Information on disease processes associated with dementia and cognitive impairment, including progression of the diseases, types and stages of memory loss, family dynamics, behavioral symptoms and limitations to normal activities of daily living;<br>(2) Information on non-pharmacological techniques and approaches used to guide and support residents with dementia/ cognitive impairment, wandering, and socially challenging behavioral expressions of need or distress;<br>(3) Information on communication techniques that facilitate

	supportive and interactive staff-resident relations;<br>(4) Positive therapeutic approaches and activities such as exercise, sensory stimulation, activities of daily living and social, recreation, and rehabilitative activities;<br>(5) Information on recognizing physical symptoms that may cause a change in dementia/cognitive impairment such as<br>dehydration, infection, and swallowing difficulty; along with individualized approaches to assist or address associated symptoms such as pain, decreased appetite and fluid intake, and/or isolation; and<br>(6) Benefits and importance of person-centered care planning and collaborative approaches to delivery of care.<br>25.16 The assisted living residence shall ensure that each staff member assigned to the secure environment is trained on the care plan for each new resident that is part of the individual's assigned duties and responsibilities.
Staff training	Arkansas. Assisted Living Facilities Level 1<br>016.25.13-504. Personnel and General Policies and Procedures<br>504.6 Dementia training shall be culturally competent for covered staff members and persons with Alzheimer's disease and dementia.<br>a. Dementia training relative to Section 504.5 shall include principles of person-centered dementia care including:<br>1. Thorough knowledge of persons with Alzheimer's disease and dementia and their abilities and needs;<br>2. Methods for ensuring optimal functioning and quality of life including how to use problem-solving approaches to care, and techniques that ensure and preserve a resident's respect, values, choice, and dignity.<br>b. The curriculum used for the initial training shall cover the following topics:<br>1. Alzheimer's disease and other dementias;<br>2. Person-centered care;<br>3. Assessment and care planning;<br>4. Activities of daily living; and<br>5. Dementia-related behaviors and communication<br>d. Dementia training for other covered staff members shall include, at a minimum:<br>1. An overview of Alzheimer's disease and other dementias;<br>2. Principles of person-centered care; and<br>3. Dementia-related communication issues<br>e. The individual providing the training shall possess:<br>1. No less than two (2) years of work experience related to Alzheimer's disease or other dementias or in health care, gerontology, or another related field;

	2. A minimum of two (2) years of general nursing experience including at least one (1) year of nursing services in a nursing facility setting or an assisted living facility within the last five (5) years; or<br>3. A minimum of two (2) years of experience as an administrator in an assisted living facility or a facility that provides direct care to persons with dementia; and<br>4. Does not have any disciplinary action regarding their license by the licensing entity or authority.
Staff training	Minnesota AL<br>144G.63. Orientation and annual training requirements<br>Subdivision 1. Orientation of staff and supervisors. All staff providing and supervising direct services must complete an orientation to assisted living facility licensing requirements and regulations before providing assisted living services to residents. The orientation may be incorporated into the training required under subdivision 5. The orientation need only be completed once for each staff person and is not transferable to another facility.<br>Subd. 2. Content of required orientation. (a) The orientation must contain the following topics:<br>(1) an overview of this chapter;<br>(2) an introduction and review of the facility's policies and procedures related to the provision of assisted living services by the individual staff person;<br>(3) handling of emergencies and use of emergency services;<br>(4) compliance with and reporting of the maltreatment of vulnerable adults under section 626.557 to the Minnesota Adult Abuse Reporting Center (MAARC);<br>(5) the assisted living bill of rights and staff responsibilities related to ensuring the exercise and protection of those rights;<br>(6) the principles of person-centered planning and service delivery and how they apply to direct support services provided by the staff person<br>144G.64 Training in dementia care required<br>(a) All assisted living facilities must meet the following training requirements:<br>(5) person-centered planning and service delivery.
Care/service planning	Nevada 449.259. Supervision and treatment of residents generally<br>1. A residential facility shall ensure that the staff of the facility collaborate with each resident of the facility, the family of the resident and other persons who provide care for the resident, including, without limitation, a qualified provider of health care, as interpreted by section 8 of this regulation, to:<br>(a) Develop a person-centered service plan for the resident; and

	(b) Review the person-centered service plan at least once each year.<br>2. A person-centered service plan developed pursuant to this section must include, without limitation:<br>(a) Provisions concerning activities of daily living, medication management, cognitive safety, assistive devices, special needs, social and recreational needs and involvement of ancillary services;<br>(b) Protective supervision as necessary for the resident;<br>(c) The manner in which all caregivers will be informed of the required supervision of the resident;<br>(d) The manner in which the facility will ensure that the resident has the opportunity to attend the religious service of his or her choice and participate in personal and private pastoral counseling;<br>(e) Permission for the resident to rest in his or her room at any time;<br>(f) Permission for the resident to enter or leave the facility at any time if the resident:<br>(1) Is physically and mentally capable of leaving the facility; and<br>(2) Complies with the rules established by the administrator of the facility for leaving the facility;<br>(g) Laundry services for the resident unless the resident elects in writing to make other arrangements;<br>(h) The manner in which the facility will ensure that the resident's clothes are clean, comfortable and presentable;<br>(i) A requirement that the facility must inform the resident or his or her representative of the actions that the resident should take to protect the resident's valuables;<br>(j) A written program of activities for the resident that includes scheduled and unscheduled activities that are suited to his or her interests and capacities; and<br>(k) If the resident has Alzheimer's disease or another form of dementia, measures to address that dementia and ensure the safety of the resident in the facility, including, without limitation:<br>(1) Any measures taken pursuant to NAC 449.2754 or 449.2756; and<br>(2) Provisions for the transfer of the resident pursuant to NAC 449.2706 if:<br>(I) It is determined through an assessment conducted pursuant to paragraph (c) of subsection 1 of NRS 449.1845 that the resident meets the criteria prescribed in paragraph (a) of subsection 2 of that section; and<br>(II) The facility does not meet the requirements of NAC 449.2754 or 449.2756 or is otherwise unable to properly care for the resident.<br>3. The administrator of a residential facility may require a resident who leaves the facility to inform a member of the staff of the facility upon his or her departure and return.

	4. The employees of a residential facility shall:<br>(a) Treat each resident in a kind and considerate manner; and<br>(b) Respect each resident's independence and ability to make decisions on his or her own, whenever possible
Care/service planning	Minnesota Assisted LIving [applies to dementia care]<br>Subd. 2. Initial reviews, assessments, and monitoring. (a) Residents who are not receiving any assisted living services shall not be required to undergo an initial nursing assessment.<br>(b) An assisted living facility shall conduct a nursing assessment by a registered nurse of the physical and cognitive needs of the prospective resident and propose a temporary service plan prior to the date on which a prospective resident executes a contract with a facility or the date on which a prospective resident moves in, whichever is earlier. If necessitated by either the geographic distance between the prospective resident and the facility, or urgent or unexpected circumstances, the assessment may be conducted using telecommunication methods based on practice standards that meet the resident's needs and reflect person-centered planning and care delivery.<br>Minnesota Assisted Living<br>144G.70 Services<br>Subd. 2. Initial reviews, assessments, and monitoring. (a) Residents who are not receiving any assisted living services shall not be required to undergo an initial nursing assessment.<br>(b) An assisted living facility shall conduct a nursing assessment by a registered nurse of the physical and cognitive needs of the prospective resident and propose a temporary service plan prior to the date on which a prospective resident executes a contract with a facility or the date on which a prospective resident moves in, whichever is earlier. If necessitated by either the geographic distance between the prospective resident and the facility, or urgent or unexpected circumstances, the assessment may be conducted using telecommunication methods based on practice standards that meet the resident's needs and reflect<br>person-centered planning and care delivery.<br>Minnesota. Assisted Living Facilities with Dementia Care<br>144G.82. Additional responsibilities of administration for assisted living facilities with dementia care<br>Subd. 3. Policies. (a) In addition to the policies and procedures required in the licensing of all facilities, the assisted living facility with dementia care licensee must develop and implement policies and procedures that address the:<br>(1) philosophy of how services are provided based upon the assisted living facility licensee's values, mission, and promotion of person-centered care and how the philosophy shall be

	implemented;<br>(2) evaluation of behavioral symptoms and design of supports for intervention plans, including nonpharmacological practices that are person-centered and evidence-informed;
Social Activities	**Oregon Assisted living**<br>**411-057-0160. Resident Services in a Memory Care Community**<br>(2) At time of move-in and quarterly, the community must make reasonable attempts to identify the preferred customary routines of each resident and the resident's preferences in how services may be delivered. Minimum services to be provided include:<br>(d) Meaningful activities that promote or help sustain the physical and emotional well-being of residents. The activities must be person centered and available during residents' waking hours.<br>(A) Each resident must be evaluated for activities according to the licensing rules of the facility. In addition, the evaluation must address the following:<br>(i) Past and current interests;<br>(ii) Current abilities and skills;<br>(iii) Emotional and social needs and patterns;<br>(iv) Physical abilities and limitations;<br>(v) Adaptations necessary for the resident to participate; and<br>(vi) Identification of activities for behavioral interventions.<br>(B) An individualized activity plan must be developed for each resident based on their activity evaluation. The plan must reflect the resident's activity preferences and needs.<br>(C) A selection of daily structured and non-structured activities must be provided and included on the resident's activity service or care plan as appropriate. Daily activity options based on resident evaluation may include but are not limited to:<br>(i) Occupation or chore related tasks;<br>(ii) Scheduled and planned events (e.g. entertainment, outings);<br>(iii) Spontaneous activities for enjoyment or those that may help diffuse a behavior;<br>(iv) One to one activities that encourage positive relationships between residents and staff (e.g. life story, reminiscing, music);<br>(v) Spiritual, creative, and intellectual activities;<br>(vi) Sensory stimulation activities;<br>(vii) Physical activities that enhance or maintain a resident's ability to ambulate or move; and<br>(viii) Outdoor activities.
Activities	Texas. Assisted Living<br>§ 553.309. Activities Program

	(a) A facility must encourage socialization, cognitive awareness, self-expression, and physical activity in a planned and structured activities program. Activities must be individualized, based upon the resident assessment, and appropriate for each resident's abilities.<br>(b) The activity program must contain a balanced mixture of activities addressing cognitive, recreational, and activity of daily living (ADL) needs.<br>(1) Cognitive activities include arts, crafts, storytelling, poetry readings, writing, music, reading, discussion, reminiscences, and reviews of current events.<br>(2) Recreational activities include all socially interactive activities, such as board games and cards, and physical exercise. Care of pets is encouraged.<br>(3) Self-care ADLs include grooming, bathing, dressing, oral care, and eating. Occupational ADLs include cleaning, dusting, cooking, gardening, and yard work. Residents must be allowed to perform self-care ADLs as long as they are able, to promote independence and self-worth.<br>(c) Residents must be encouraged, but never forced, to participate in activities. Residents who choose not to participate in a large group activity must be offered at least one small group or one-on-one activity per day.<br>(d) Facilities must have an employee responsible for leading activities.<br>(1) Facilities with 16 or fewer residents must designate an employee to plan, supply, implement, and record activities.<br>(2) Facilities with 17 or more residents must employ, at a minimum, an activity director for 20 hours weekly. The activity director must be a qualified professional who:<br>(A) is a qualified therapeutic recreation specialist or an activities professional who is eligible for certification as a therapeutic recreation specialist, a therapeutic recreation assistant, or an activities professional by a recognized accrediting body, such as the National Council for Therapeutic Recreation Certification, the National Certification Council for Activity Professionals, or the Consortium for Therapeutic Recreation/Activities Certification, Inc.;<br>(B) has two years of experience in a social or recreational program within the last five years, one year of which was full-time in an activities program in a health care setting; or<br>(C) has completed an activity director training course approved by the National Association for Activity Professionals or the National Therapeutic Recreation Society.<br>(e) The activity director or designee must review each resident's medical and social history, preferences, and dislikes, in determining appropriate activities for the resident. Activities must be tailored to each resident's unique requirements and skills.

	(f) The activities program must provide opportunities for group and individual settings. On weekdays, each resident must be offered at least one cognitive activity, two recreational activities, and three ADL activities each day. The cognitive and recreational activities (structured activities) must be at least 30 minutes in duration, with a minimum of six and a half hours of structured activity for the entire week. At least an hour and a half of structured activities must be provided during the weekend and must include at least one cognitive activity and one physical activity.<br>(g) The activity director or designee must create a monthly activities schedule. Structured activities should occur at the same time and place each week to ensure a consistent routine within the facility.<br>(h) The activity director or designee must annually attend at least six hours of continuing education regarding Alzheimer's disease or related disorders.<br>(i) Special equipment and supplies necessary to accommodate persons with a physical disability or other persons with special needs must be provided as appropriate.
**Family-involved care**	
Care/service planning	Georgia Personal Care Home 111-8-62-.19 and Assisted Living 111-8-63-.19 Additional Requirements for Certified Memory Care Centers<br>Resident Needs Assessment. The assisted living community must complete an assessment of the resident that addresses the resident's care needs taking into account the resident's family supports, the resident's functional capacity relative to the activities of daily living, physical care needs, medical information provided, cognitive and behavioral impairments, if any, and personal preferences relative to care needs.<br>Written Care Plan. Utilizing the information acquired during the admission process and the move-in adjustment period, a home which provides proxy caregivers or memory care must develop the resident's individual written care plan within 14 days of admission and require staff to use the care plan as a guide for the delivery of care and services to the resident. The<br>care plan must include the following:<br>(a) A description of the resident's care and social needs and the services to be provided, including frequency to address care and social needs.<br>(b) Resident's particular preferences regarding care, activities and interests.<br>(c) Specific behaviors to be addressed with interventions to be used.

	(d) Any physician order or order of a nurse practitioner or physician assistant working under protocol or job description, respectively for assistive devices.<br>(e) Staff primarily responsible for implementing the care plan.<br>(f) Evidence of resident and family involvement in the development of the plan when appropriate a home which provides proxy caregivers or memory care must develop the resident's individual written care plan within 14 days of admission and require staff to use the care plan as a guide for the delivery of care and services to the resident. The care plan must include the following:<br>(a) A description of the resident's care and social needs and the services to be provided, including frequency to address care and social needs.<br>(b) Resident's particular preferences regarding care, activities and interests.<br>(c) Specific behaviors to be addressed with interventions to be used.<br>(d) Any physician order or order of a nurse practitioner or physician assistant working under protocol or job description, respectively for assistive devices.<br>(e) Staff primarily responsible for implementing the care plan.<br>(f) Evidence of resident and family involvement in the development of the plan when appropriate involvement in the development of the plan, if possible, with incorporation of family and personal history to support a person-centered approach to care.<br>(8) Post-Admission Assessment. The home must assess each resident's care needs to include the following components: resident's family supports, level of activities of daily living functioning, physical care needs and level of behavior impairment.<br>Additional Requirements for Certified Memory Care Centers<br>(2) Written Description. The home must develop an accurate written description of the memory care center that includes the following: ... (l)The role of the family in caring for residents with Alzheimer's and other dementias.
Care/service planning	Florida. Assisted Living Residences<br>59A-36.021. Extended Congregate Care Services.<br>(2) EXTENDED CONGREGATE CARE POLICIES. Policies and procedures established through extended congregate care services must promote resident independence, dignity, choice, and decision-making. The facility must develop and implement specific written policies and procedures that address:<br>(g) How to involve residents in decisions concerning the resident. The services must provide opportunities and encouragement for the resident to make personal choices and decisions. If a resident

	needs assistance to make choices or decisions, a family member or other resident representative must be consulted. Choices must include at a minimum whether:<br>1. To participate in the process of developing, implementing, reviewing, and revising the resident's service plan,<br>2. To remain in the same room in the facility, except that a current resident transferring into an extended congregate care services may be required to move to the part of the facility licensed for extended congregate care, if only part of the facility is so licensed,<br>3. To select among social and leisure activities,<br>4. To participate in activities in the community. At a minimum the facility must arrange transportation to such activities if requested by the resident; and,<br>5. To provide input with respect to the adoption and amendment of facility policies and procedures.<br>59A-36.021. Extended Congregate Care Services.<br>(c) Pursuant to the definitions of “shared responsibility” and “managed risk” as provided in Section 429.02, F.S., the service plan must be developed and agreed upon by the resident or the resident's representative or designee, surrogate, guardian, or attorney-in-fact, and must reflect the responsibility and right of the resident to consider options and assume risks when making choices pertaining to the resident's service needs and preferences.
Care/service planning	Georgia Personal Care Home<br>111-8-62-.19. Additional Requirements for Certified Memory Care Centers.<br>(1) In addition to all other requirements contained in this Chapter, where an assisted living community holds itself out as providing additional or specialized care to persons with probable diagnoses of Alzheimer's Disease or other dementia, or charges rates in excess of that charged other residents for the provision of additional or specialized care, the assisted living community must meet the following requirements:<br>(g) Individual Written Care Plan and Reviews. The resident's written care plan will be developed or updated by staff with at least one member of the specialized memory care staff providing direct care participating. Input from each shift of direct care staff that provides care to the resident will be requested. All team members participating shall sign the written care plan and the plan will be shared with the direct care staff providing care to the resident and serve as a guide for the delivery of care to the resident. The resident's family shall participate in the development of the plan, if possible, with incorporation of family and personal history to support a person-centered approach to care. The written care plan must be reviewed at least quarterly and modified as changes in the

	resident's needs occur.<br>Georgia Assisted Living Community,<br>111-8-63-.17. Services in the Community.<br>(3) Written Care Plan. Utilizing the information acquired during the admission process and the move-in adjustment period, the assisted living community must develop the resident's individual written care plan within 14 days of admission and require staff to use the care plan as a guide for the delivery of care and services to the resident. The care plan must include the following:<br>(e) Evidence of family involvement in the development of the plan, if possible, with incorporation of family and personal history to support a person-centered approach to care.<br>Resident Needs Assessment. The assisted living community must complete an assessment of the resident that addresses the resident's care needs taking into account the resident's family supports, the resident's functional capacity relative to the activities of daily living, physical care needs, medical information provided, cognitive and behavioral impairments, if any, and personal preferences relative to care needs.<br>Evidence of resident and family involvement in the development of the plan when appropriate
Family assistance with care	Washington 388-78A-2290.<br>Family assistance with medications and treatments.<br>(1) An assisted living facility may permit a resident's family member to administer medications or treatments or to provide medication or treatment assistance, including obtaining medications or treatment supplies, to the resident.<br>(2) The assisted living facility must disclose to the department, residents, the residents' legal representatives, if any, and if not the residents' representative if any, and to interested consumers upon request, information describing whether the assisted living facility permits such family administration or assistance and, if so, the extent of any limitations or conditions.<br>(3) If the assisted living facility allows family assistance with or administration of medications and treatments, and the resident and a family member(s) agree a family member will provide medication or treatment assistance, or medication or treatment administration to the resident, the assisted living facility must request that the family member submit to the assisted living facility a written plan for such assistance or administration that includes at a minimum:<br>(a) By name, the family member who will provide the medication or treatment assistance or administration;<br>(b) A description of the medication or treatment assistance or administration that the family member will provide, to be referred to as the primary plan;

	(c) An alternate plan if the family member is unable to fulfill his or her duties as specified in the primary plan;<br>(d) An emergency contact person and telephone number if the assisted living facility observes changes in the resident's overall functioning or condition that may relate to the medication or treatment plan; and<br>(e) Other information determined necessary by the assisted living facility.<br>(4) The plan for family assistance with medications or treatments must be signed and dated by:<br>(a) The resident, if able;<br>(b) The resident's representative, if any;<br>(c) The resident's family member responsible for implementing the plan; and<br>(d) A representative of the assisted living facility authorized by the assisted living facility to sign on its behalf.<br>(5) The assisted living facility may, through policy or procedure, require the resident's family member to immediately notify the assisted living facility of any changes in the medication or treatment plans for family assistance or administration.<br>(6) The assisted living facility must require that whenever a resident's family provides medication assistance or medication administration services, the resident's significant medications remain on the assisted living facility premises whenever the resident is on the assisted living facility premises.<br>(7) The assisted living facility's duty of care shall be limited to: Observation of the resident for changes in overall functioning consistent with RCW 18.20.280; notification to the person or persons identified in RCW 70.129.030 when there are observed changes in the resident's overall functioning or condition, or when the assisted living facility is aware that both the primary and alternate plan are not implemented; and appropriately responding to obtain needed assistance when there are observable or reported changes in the resident's physical or mental functioning.
Family assistance with care	Florida. 59A-36.021. Extended Congregate Care Services<br>(7) EXTENDED CONGREGATE CARE SERVICES. All services must be provided in the least restrictive environment, and in a manner that respects the resident's independence, privacy, and dignity.<br>(a) A facility providing extended congregate care services may provide supportive services including social service needs, counseling, emotional support, networking, assistance with securing social and leisure services, shopping service, escort service, companionship, family support, information and referral, assistance in developing and implementing self-directed activities, and

	volunteer services. Family or friends must be encouraged to provide supportive services for residents. The facility must provide training for family or friends to enable them to provide supportive services in accordance with the resident's service plan.
Family council	New York. Assisted Living Residences<br>Section 1000.8. Consumer and resident protections<br>(d) Family organizations.<br>(1) The operator shall encourage and assist residents' families and representatives who so desire to organize and maintain committees, councils, or such other self-governing body as the residents' families and representatives may choose.<br>(2) The operator shall:<br>(i) assist the residents' family and representative organization:<br>(a) in meeting as often as the membership deems necessary;<br>(b) in assuring the organization is chaired and directed by the residents' families and/or representatives; and<br>(c) in meeting with any member of the supervisory staff, provided that reasonable notice of the request is given to such staff;<br>(ii) appoint a staff person to act as an advisor to the residents' family and representative organization, who shall serve as a liaison between the organization and administration to report all problems, issues and suggestions discussed by the families and representatives which require administrative action; and<br>(iii) assure that any complaints, problems or issues reported by the residents' family and representative organization, if formed, to the designated staff person or administration are addressed, and that a written report addressing the problems, issues or suggestions is sent to the organization;<br>(iv) assure that the residents' family and representative organization, if formed, have the freedom to meet without interference and be provided space to conduct such meetings.<br>(e) Grievances and recommendations. The operator shall develop written procedures and shall establish and maintain a system to receive and respond, within 21 days of receipt, to grievances and recommendations for change or improvement in residence operations and programs which are presented by residents and their family and representatives, in accordance with 18 NYCRR sections 487.5(c) and 488.5(b)
